# Supplementary material for: Effect of Conformational Diversity on the Bioactivity of µ-Conotoxin PIIIA Disulfide Isomers
Source: Mar Drugs. 2019 Jul 2;17(7):390. doi: 10.3390/md17070390 (PMC6669574; doi:10.3390/md17070390)
Supplement: Supplementary file 1 [file marinedrugs-17-00390-s001.pdf]

Supplementary Material

Effect of conformational diversity on the bioactivity of μ-conotoxin PIIIA disulfide isomers

*Ajay Abisheck Paul George,[1‡] Pascal Heimer[1‡] Enrico Leipold,[2‡] Thomas Schmitz,[1] Desiree Kaufmann,[3] Daniel Tietze,[3] Stefan H. Heinemann,[4] and Diana Imhof[1]**

[1]Pharmaceutical Biochemistry and Bioanalytics, Pharmaceutical Institute, University of Bonn, An der Immenburg 4, D-53121 Bonn, Germany

[2]Department of Anesthesiology and Intensive Care, University of Lübeck, Ratzeburger Allee 160, D-23562 Lübeck, Germany

[3]Eduard Zintl Institute of Inorganic and Physical Chemistry, Darmstadt University of Technology, Alarich-Weiss-Str. 4, D-64287 Darmstadt, Germany

[4]Center for Molecular Biomedicine, Department of Biophysics, Friedrich Schiller University Jena and Jena University Hospital, Hans-Knöll-Str. 2, D-07745 Jena, Germany

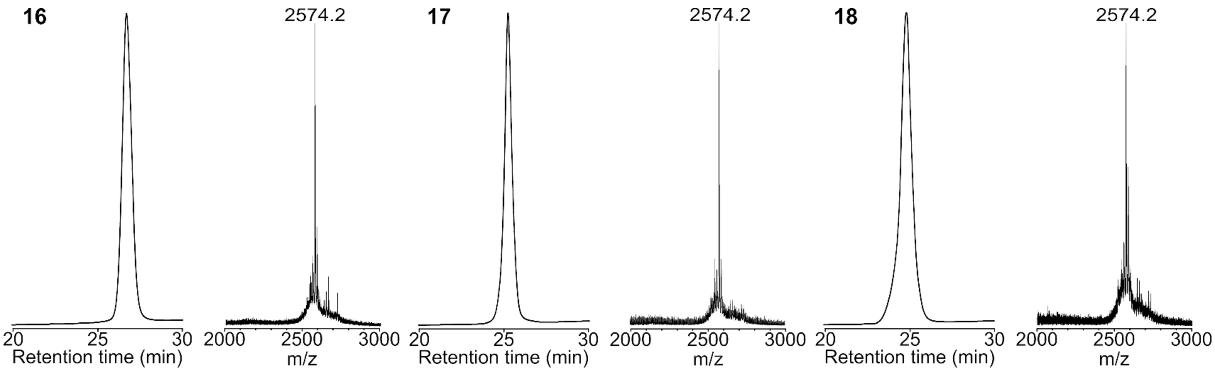

Figure S1. Peptide chemical analysis. HPLC-elution profiles (*left*, C18 column) of the oxidized and purified Ser-mutant isomers **16-18** with the corresponding MALDI MS spectra (*right*).

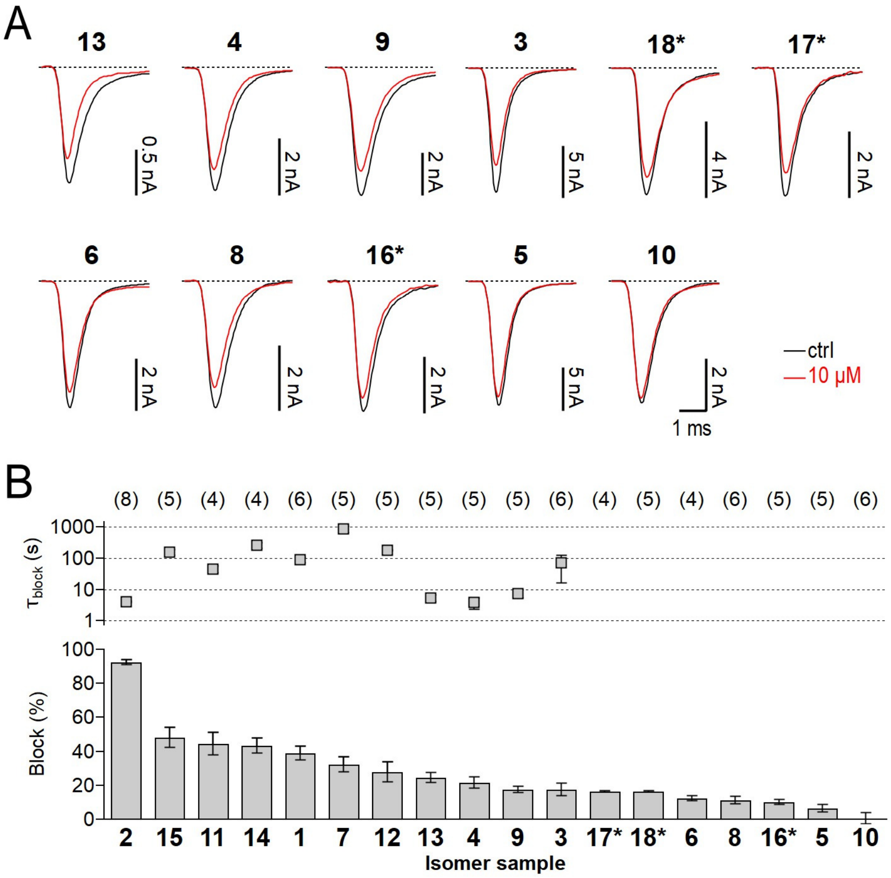

Figure S2. Block of Na$_V$1.4-mediated currents by disulfide isomers of μ-PIIIA. (A) Representative current traces of transiently expressed Na$_V$1.4 channels evoked at a test potential of –20 mV before (*black*, ctrl) and after (*red*) application of 10 μM of the indicated μ-PIIIA isomers. (B) Histogram showing steady-state block of Na$_V$1.4-mediated currents by 10 μM of the indicated μ-PIIIA isomers (bottom) and the associated single-exponential time constant, τ_{block}, describing the kinetics of channel inhibition. Numbers of individual experiments, n, are provided in parentheses. 2-Disulfide-bonded mutant isomers are marked with an asterisk.

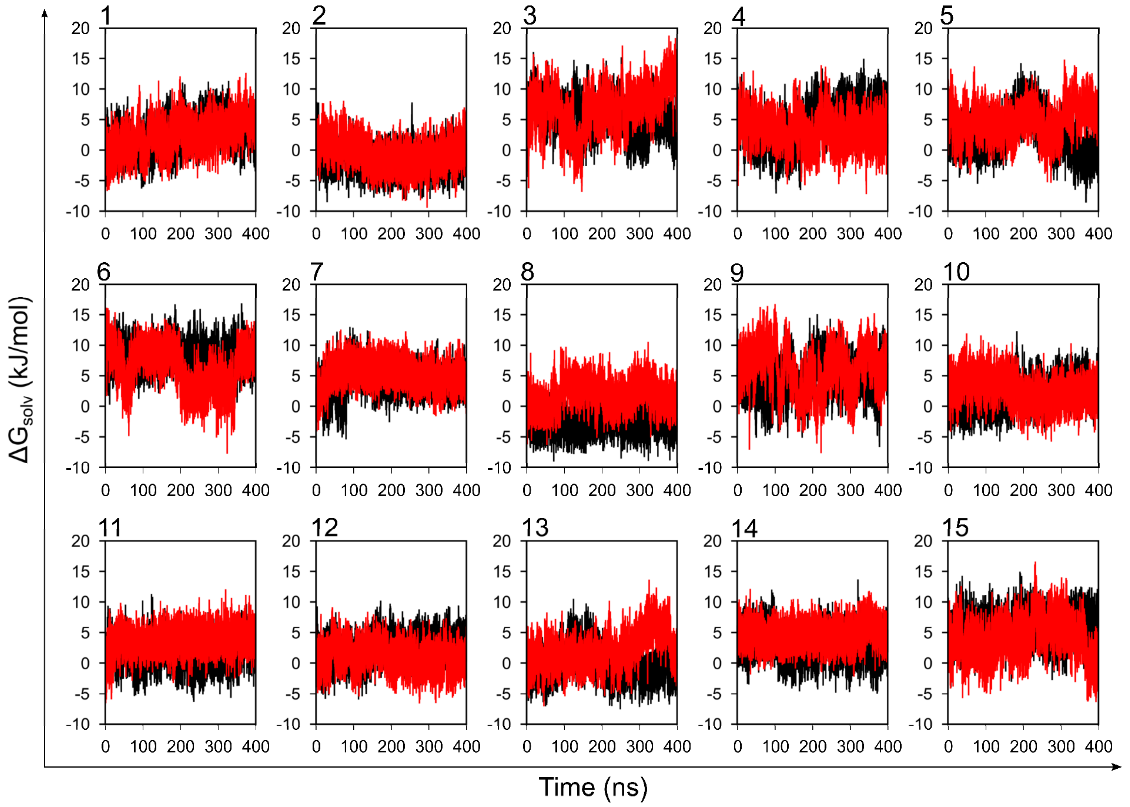

Figure S3. Solvation free energy (ΔG_{solv}) plots of the 15 μ-PIIIA disulfide isomers. Panels **1-15** feature the ΔG_{solv} (solvation free energy) plots of μ-PIIIA isomers from two independent 400-ns MD simulations of their NMR structures or models (the latter for isomers 7, 12 and 13). The black and red curves in each panel represent simulations 1 and 2.

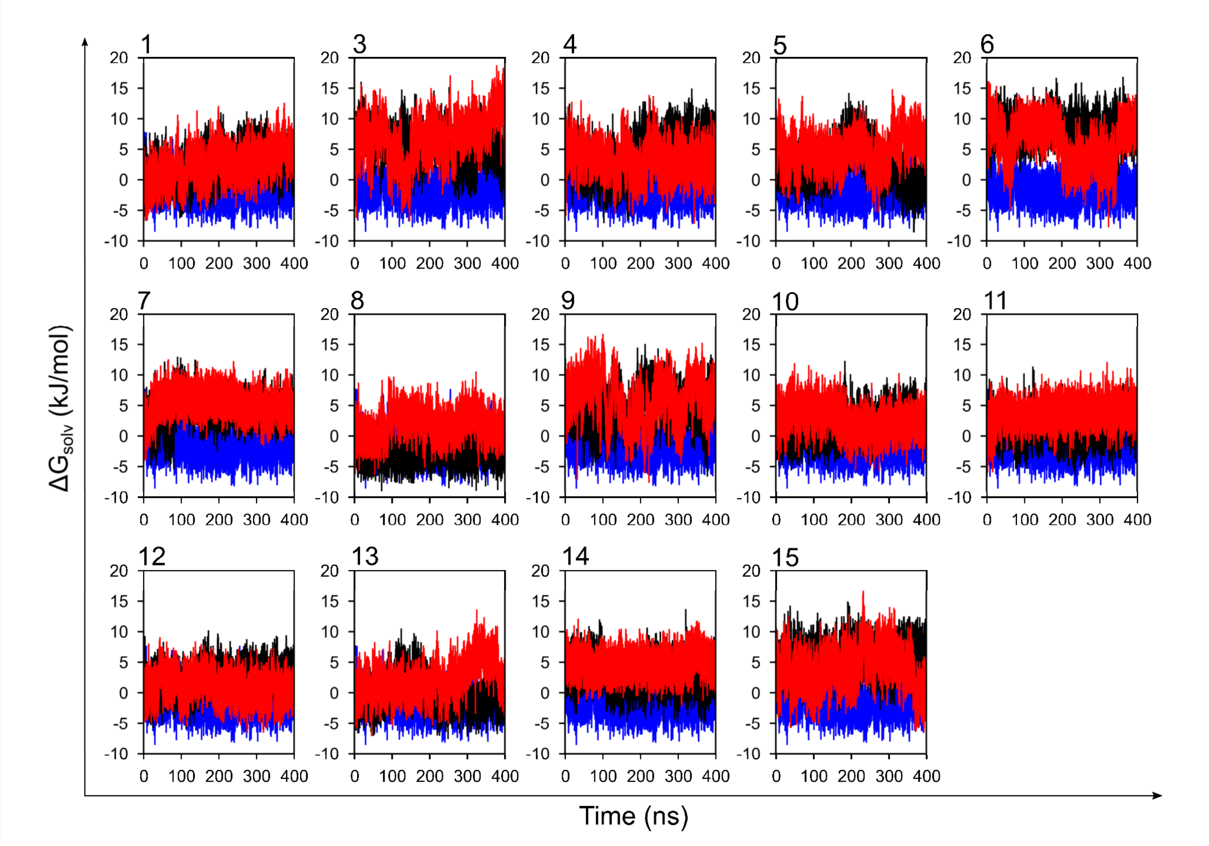

Figure S4. Comparison of solvation free energy (ΔG_{solv}) plots of the 14 non-native μ-PIIIA disulfide isomers against the native isomer 2. Panel 1 represents isomer **1.** The following panels 3-15 represent isomers **3-15**. In all panels, the blue curve represents the solvation free energy (ΔG_{solv}) plotted as a function of simulation time of the native isomer **2** as reference, while the red and blue curves represent ΔG_{solv} computed from runs 1 and 2 of two independent 400-ns MD simulations.

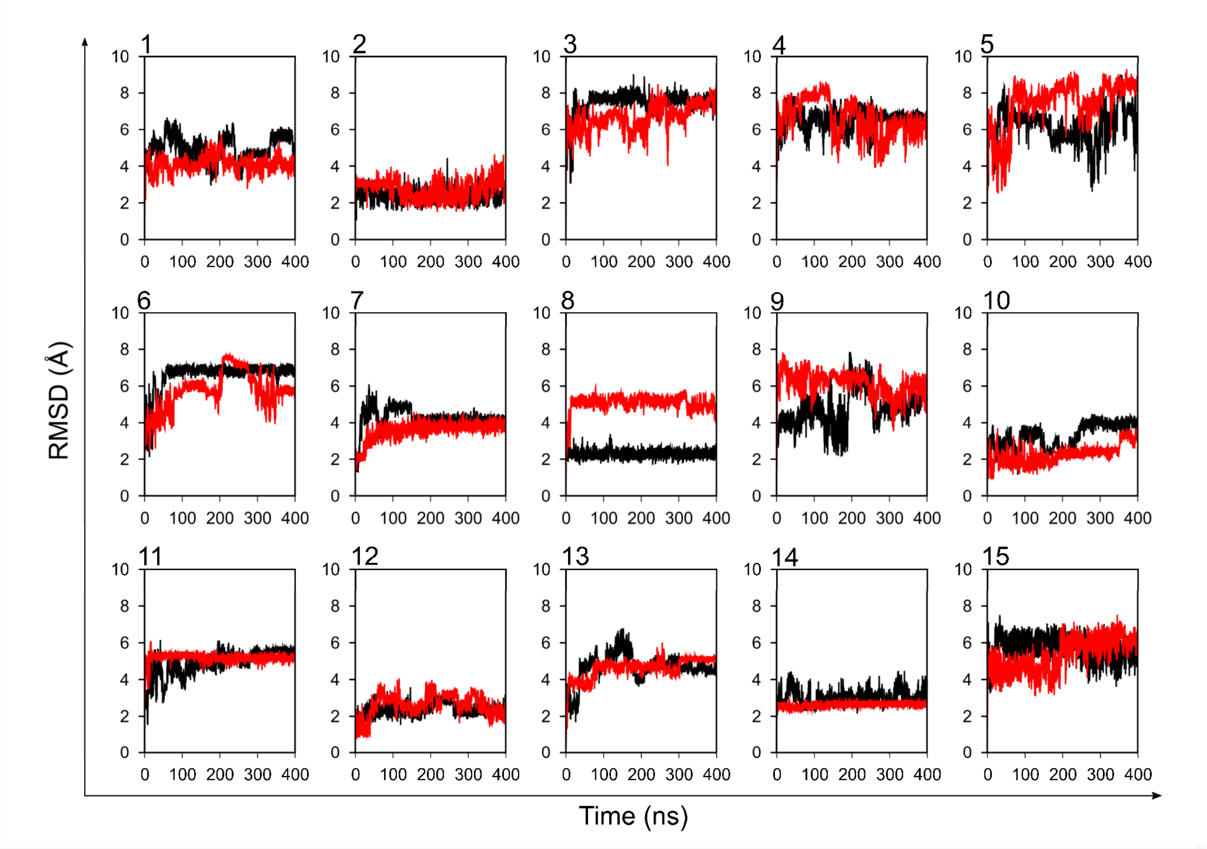

Figure S5. Backbone RMSD plots of the 15 µ-PIIIA disulfide isomers. Panels 1-15 contain the backbone RMSD (root mean squared deviation) plots of µ-PIIIA isomers **1-15** from two independent 400-ns MD simulations of their NMR structures. The black and red curves in each panel represent simulations 1 and 2.

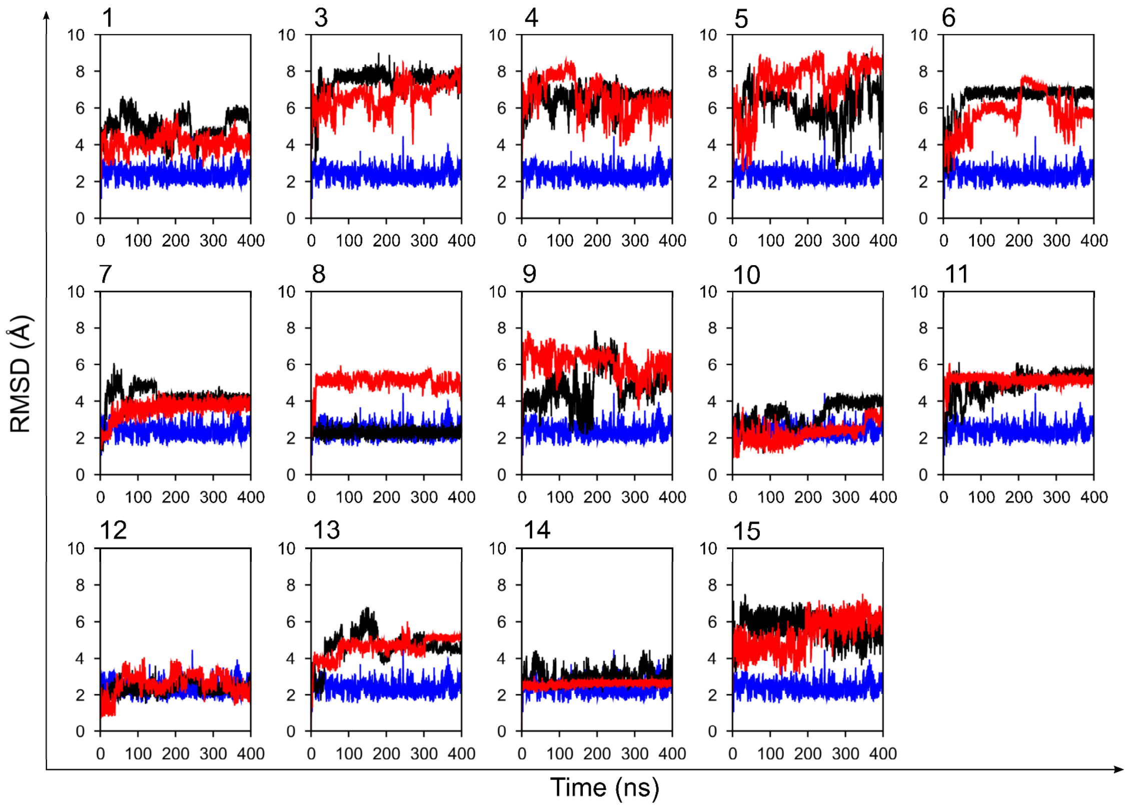

Figure S6. Comparison of backbone RMSD plots of the 14 non-native µ-PIIIA disulfide isomers against the native isomer 2. Panel 1 represents data for isomer **1.** The following panels 3-15 represent data for isomers **3-15**. In all panels, the blue curve represents the backbone RMSD of the native isomer **2** as reference, while the red and black curves represent RMSD computed from runs 1 and 2 of two independent 400-ns MD simulations for the particular isomer.

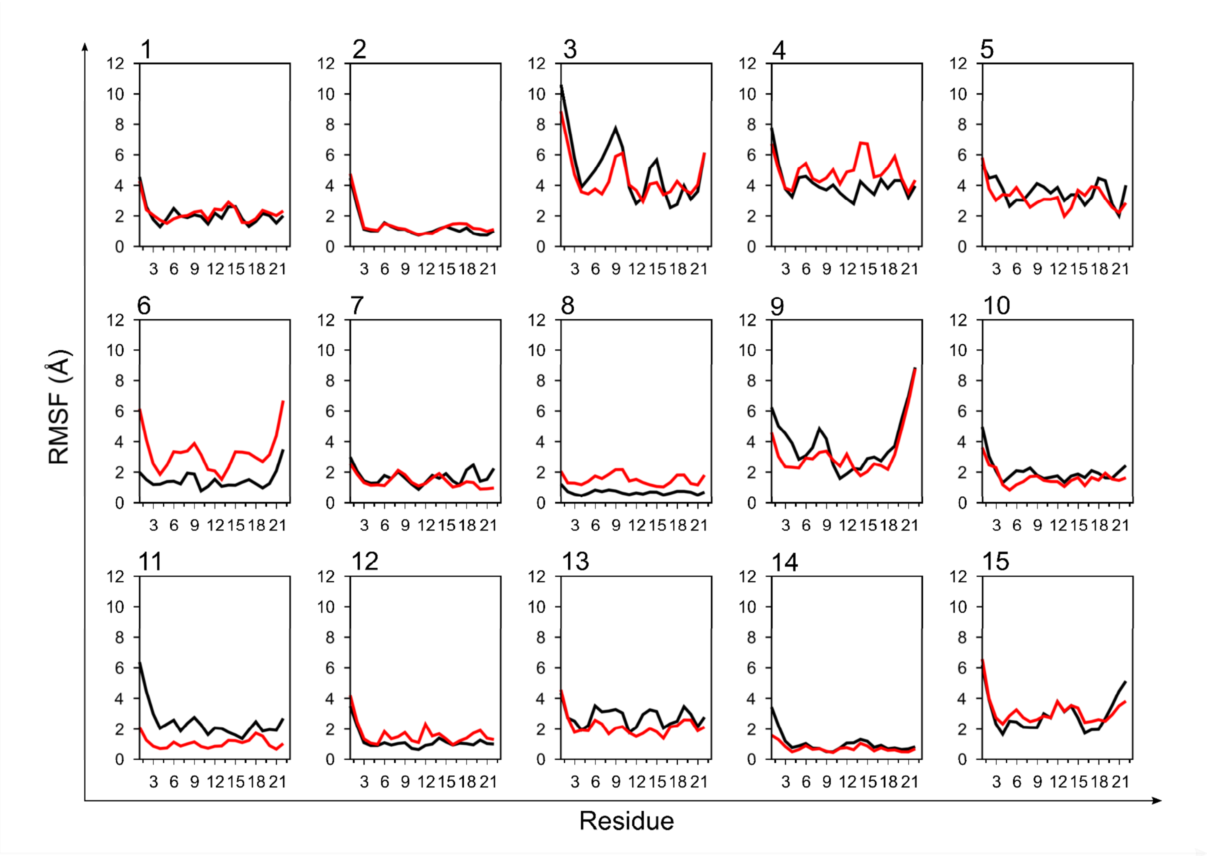

Figure S7. Per-residue RMSF plots of the 15 µ-PIIIA disulfide isomers. Panels 1-15 contain the per-residue RMSF (root mean squared fluctuation) plots of µ-PIIIA isomers **1-15** from two independent 400-ns MD simulations of their NMR structures. The black and red curves represent simulations 1 and 2, respectively. The X-axis in all panels holds the residue number of the 22 amino acid long peptide µ-PIIIA. The sequence of µ-PIIIA is ZRLCCGFOKSCRSRQCKOHRCC.

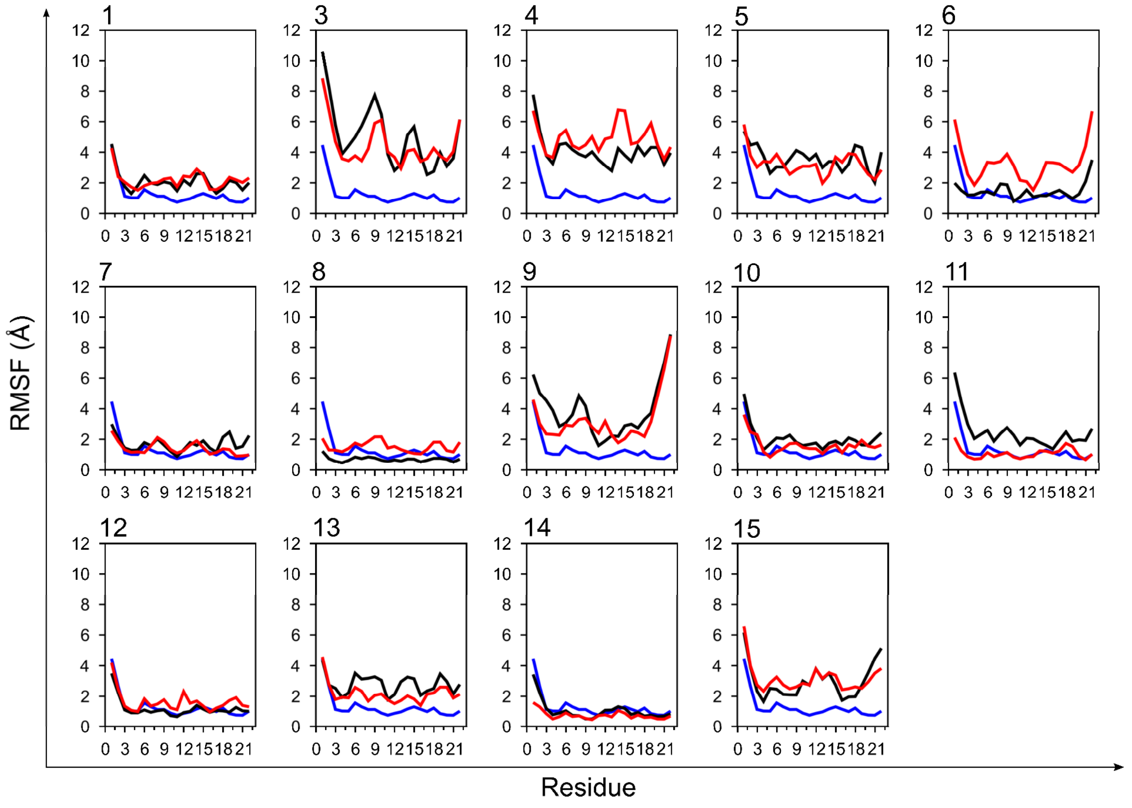

Figure S8. Comparison of the per-residue RMSF plots of the 14 non-native μ-PIIIA disulfide isomers against the native isomer 2. Panel A represents isomer **1** and the following panels B-N represent isomers **3**-**15**. In all panels, the blue curve represents the backbone the per residue RMSF of the native isomer **2** as reference, while the red and black curves represent RMSF computed from runs 1 and 2 of two independent 400-ns MD simulations for the particular isomer. The X-axes in all panels hold the residue numbers of the 22mer peptide μ-PIIIA: ZRLCCGFOKSCRSRQCKOHRCC.

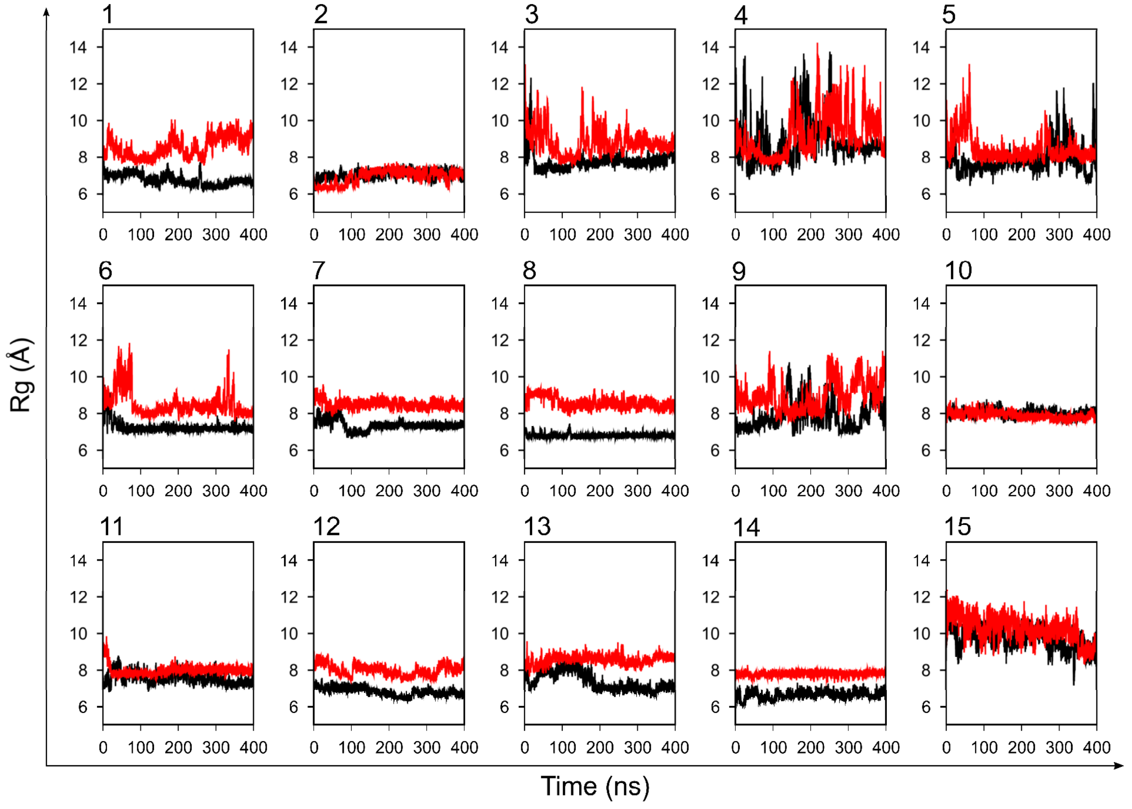

Figure S9. Rg (radius of gyration) plots of the 15 μ-PIIIA disulfide isomers. Panels 1-15 contain the Rg (radius of gyration) plots of μ-PIIIA isomers **1-15** respectively from two independent 400-ns MD simulations of their NMR structures. The black and red curves in each panel represent simulations 1 and 2.

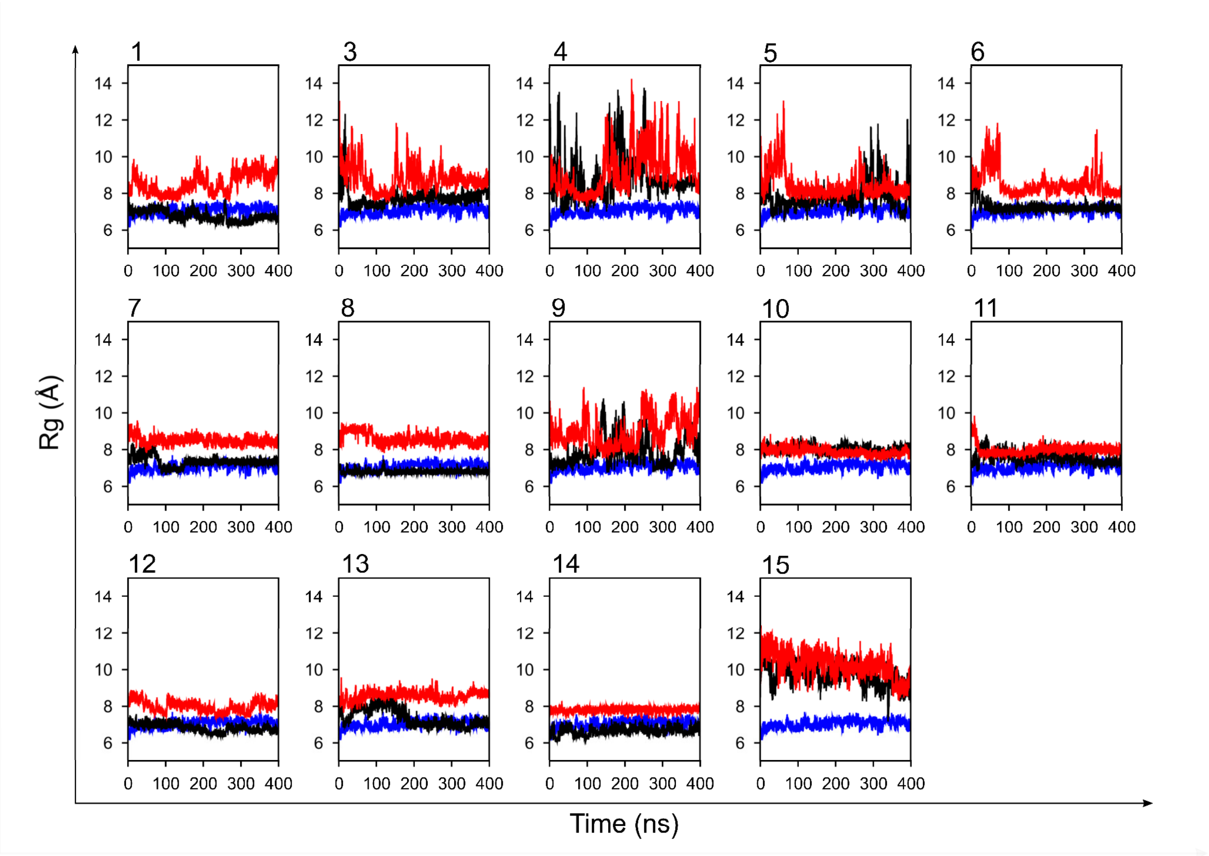

Figure S10. Comparison of Rg (radius of gyration) plots of the 14 non-native μ-PIIIA disulfide isomers against the native isomer 2. Panel 1 represents data for isomer **1** and the following panels 3-15 represent isomers **3**-**15**. In all panels, the blue curve represents the peptide Rg of the native isomer **2** as reference, while the red and black curves represent the peptide Rg computed from runs 1 and 2 of two independent 400-ns MD simulations for the particular isomer.

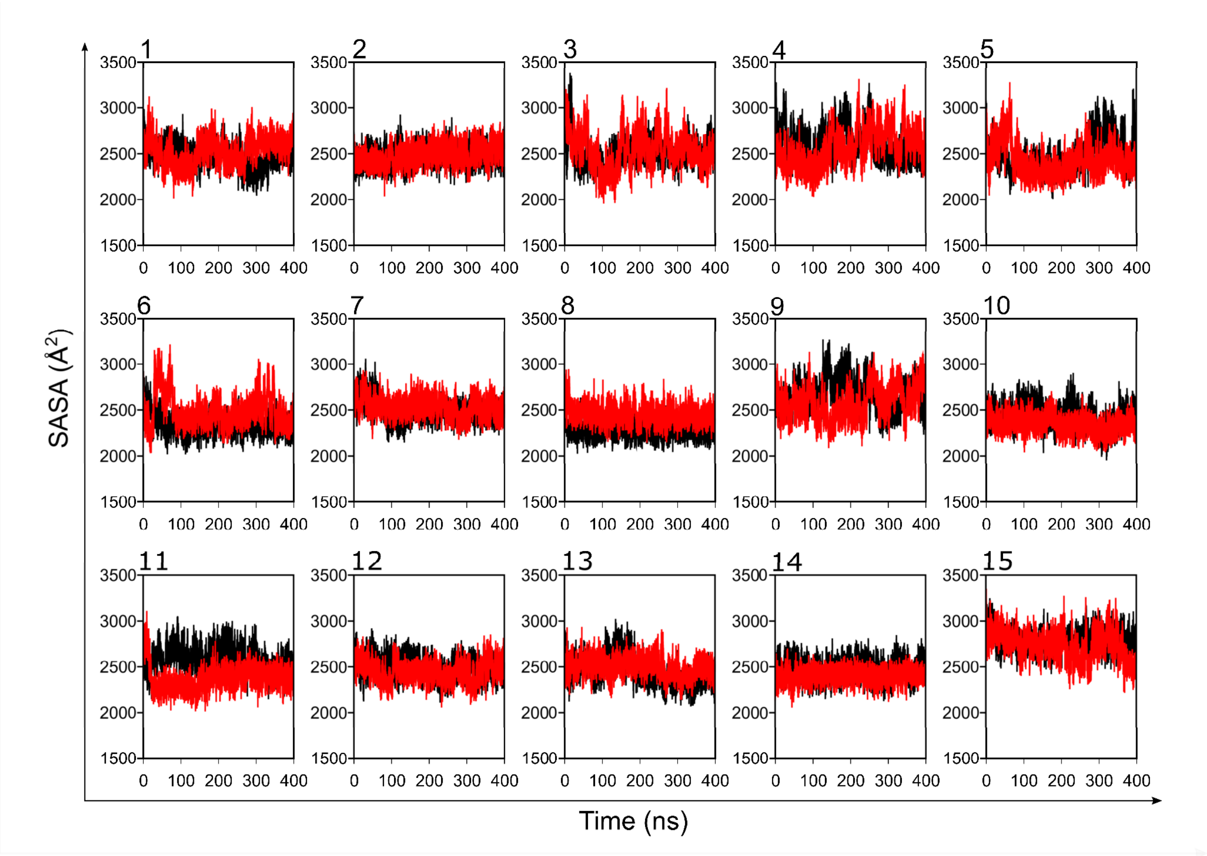

Figure S11. SASA (solvent accessible surface area) plots of the 15 μ-PIIIA disulfide isomers. Panels 1-15 contain the SASA (solvent accessible surface area) plots of μ-PIIIA isomers **1-15** from two independent 400-ns MD simulations of their NMR structures. The black and red curves in each panel arose from simulations 1 and 2.

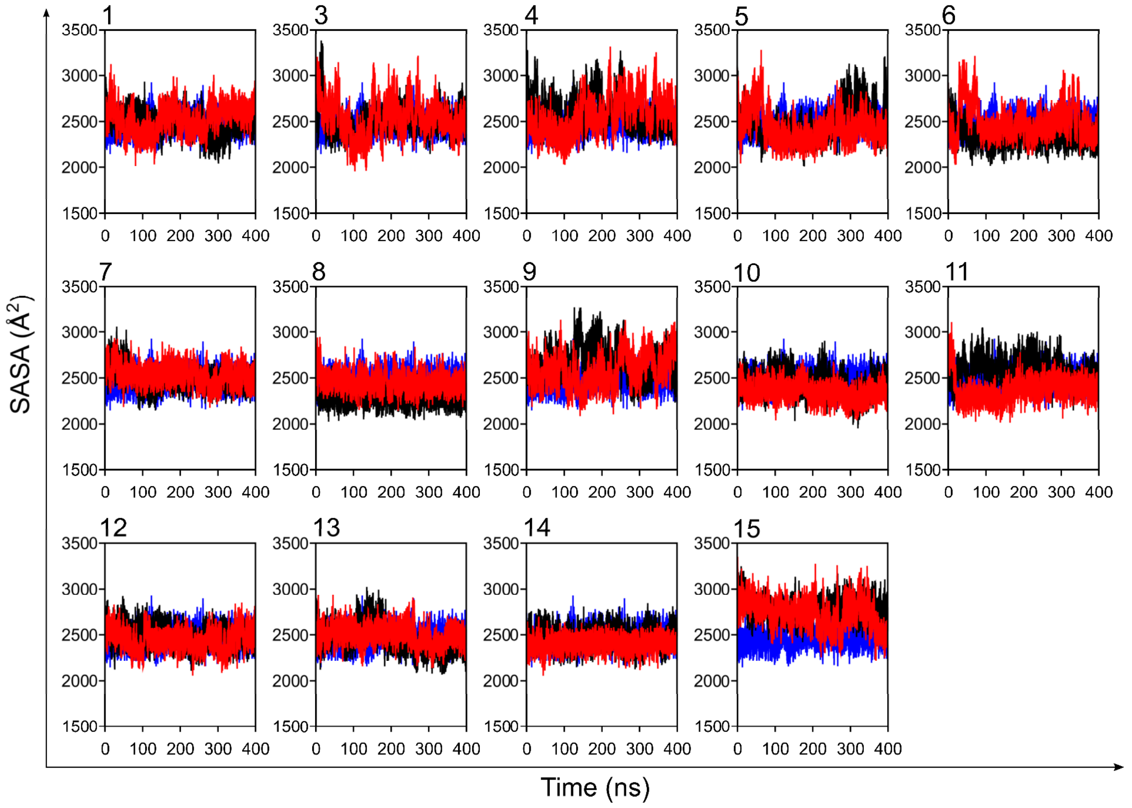

Figure S12. Comparison of SASA (solvent accessible surface area) plots of the 14 non-native μ-PIIIA disulfide isomers against the native isomer 2. Panel 1 represents data from isomer **1.** The following panels 3-15 represent data from isomers **3-15**. In all panels, the blue curve represents the peptide SASA of the native isomer **2** as reference, while the red and black curves represent the peptide SASA computed from runs 1 and 2 of two independent 400-ns MD simulations for the particular isomer.

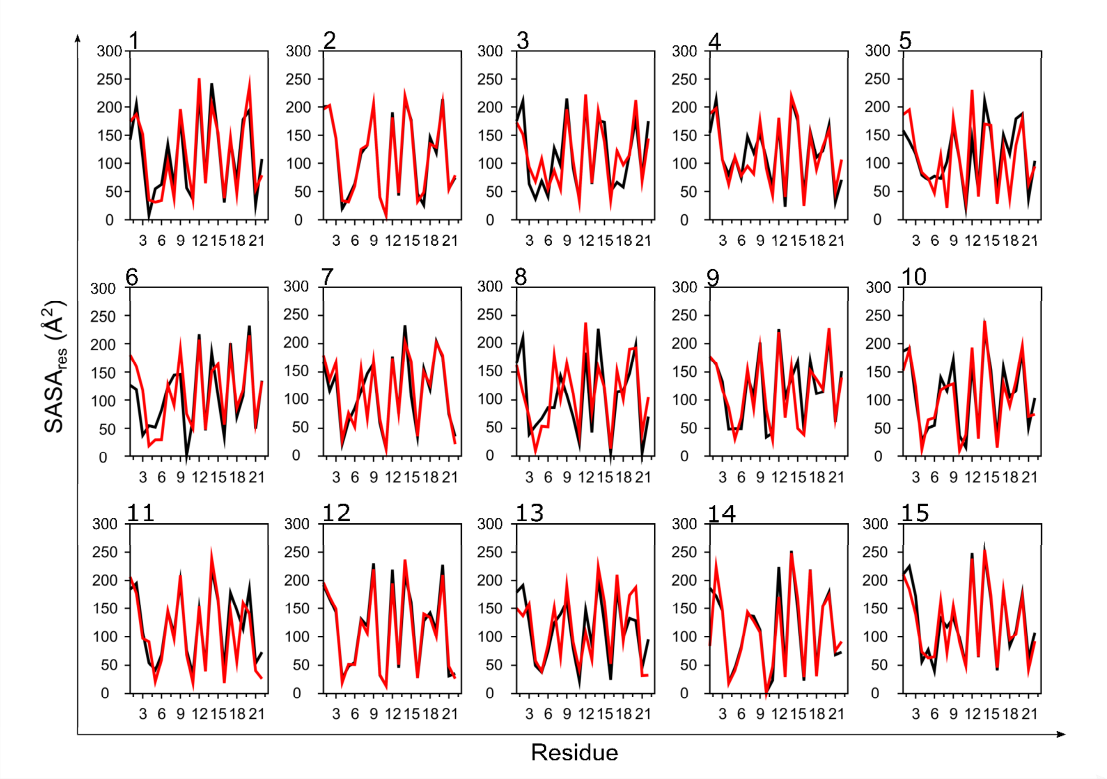

Figure S13. Per residue contribution to the solvent accessible surface area $SASA_{res}$ plots of the 15 μ-PIIIA disulfide isomers. Panels 1-15 contain the $SASA_{res}$ (per residue contribution to the solvent accessible surface area) plots of μ-PIIIA isomers **1-15** from two independent 400-ns MD simulations of their NMR structures. The black and red curves in each panel represent simulations 1 and 2. The X-axes in all panels hold the residue number of the 22mer peptide μ-PIIIA: ZRLCCGFOKSCRSRQCKOHRCC.

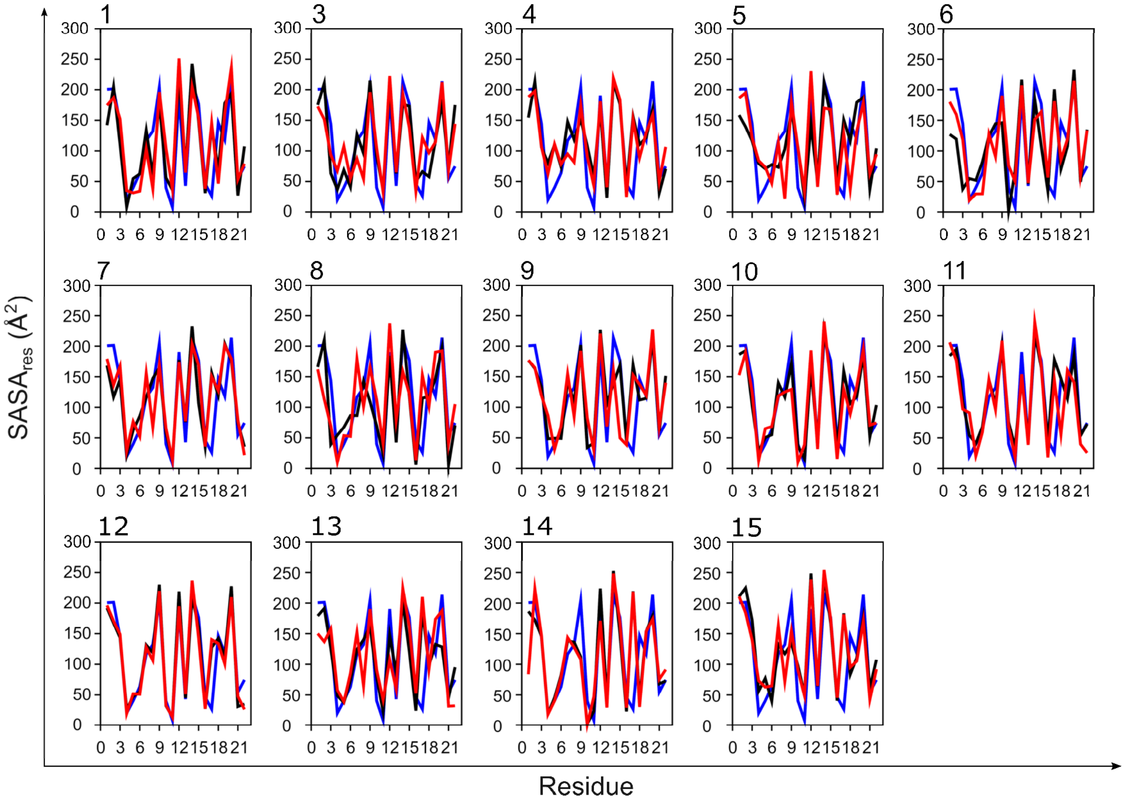

Figure S14. Comparison of SASA$_{res}$ (per residue contribution to the solvent accessible surface area) plots of the 14 non-native µ-PIIIA disulfide isomers against the native isomer 2. Panel 1 represents data from isomer **1.** The following panels 3-15 represent isomers **3-15**. In all panels, the blue curve represents the peptide SASA$_{res}$ of the native isomer **2** as reference, while the red and black curves represent the peptide SASA$_{res}$ computed from runs 1 and 2 of two independent 400-ns MD simulations for the particular isomer. The X-axes in all panels hold the residue number of the 22mer peptide µ-PIIIA: ZRLCCGFOKSCRSRQCKOHRCC.

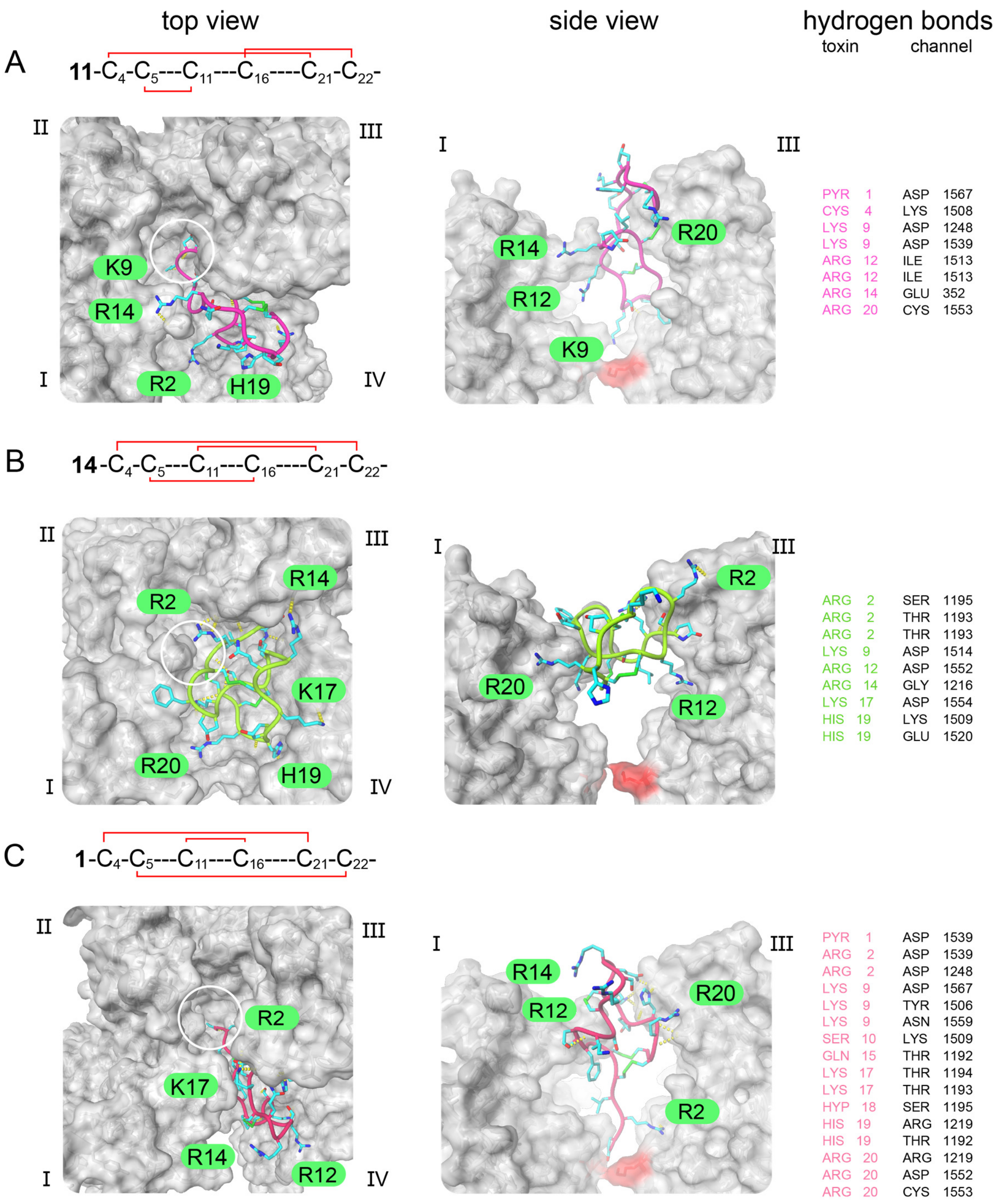

Figure S15. Visualization μ-PIIIA–Nav1.4 complex conformations obtained from docking experiments for A) the isomer 11 and B) isomer 14 and C) isomer 1. Left panel – top view of the toxin-channel complex. Middle panel – side view of the toxin-channel complex. The four $Na_V1.4$ domains are indicated; hydrogen bonds between the toxin and the channel are shown as yellow dashed lines and are specified in the right panel. The $Na_V1.4$ channel surface (molecular surface) is illustrated in gray, the selectivity filter motif (DEKA) is highlighted in red. The toxin is shown with

side-chain atoms present (coloring scheme: carbon – cyan, nitrogen – blue, oxygen – red, sulfur – green, backbone – isomer 11: pink, 14: green, 1: rosé).

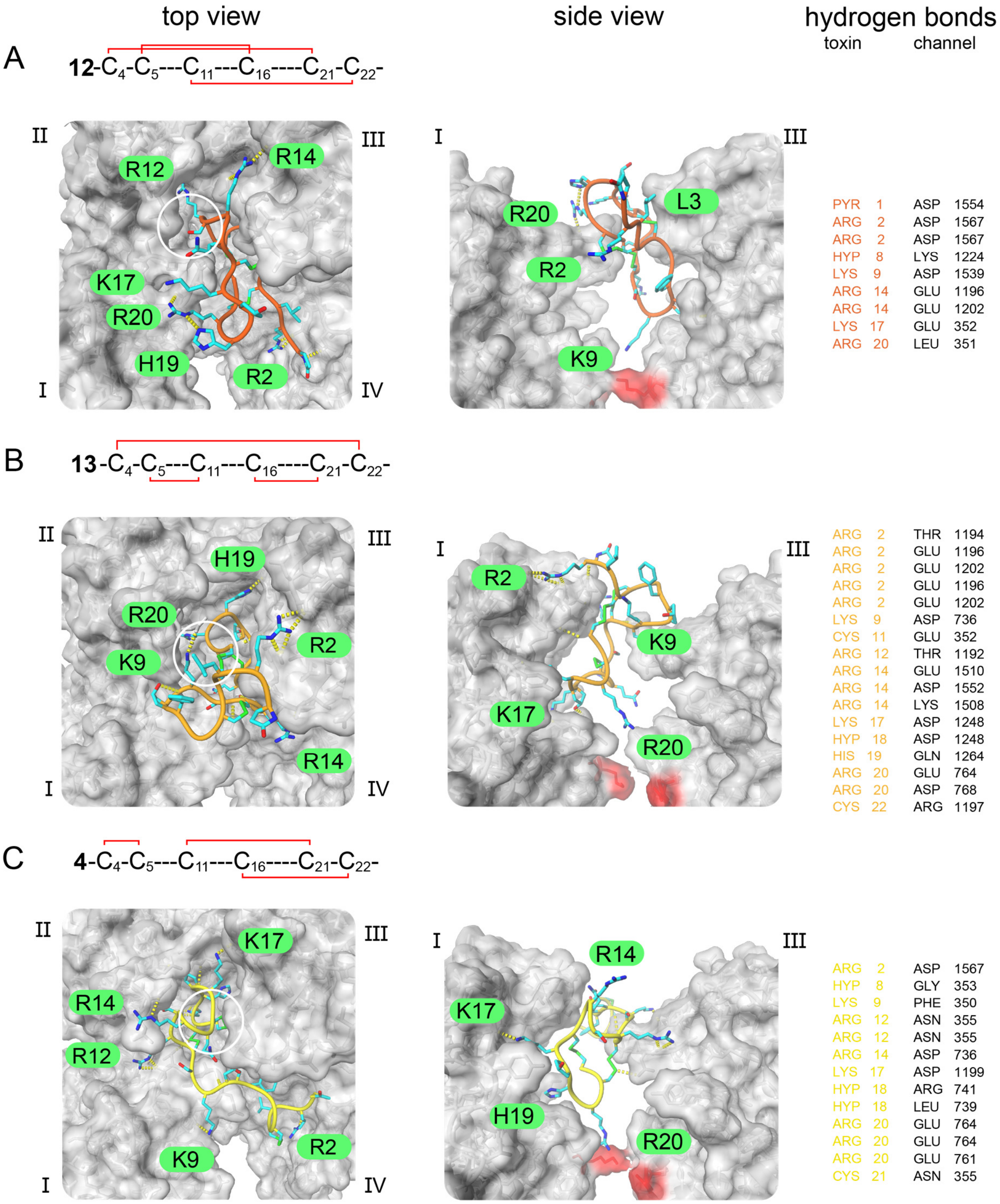

Figure S16. Visualization μ-PIIIA–Nav1.4 complex conformations obtained from docking experiments for A) the isomer 12 and B) isomer 13 and C) isomer 4. Left panel – top view of the toxin-channel complex. Middle panel – side view of the toxin-channel complex. The four $Na_V1.4$ domains are indicated; hydrogen bonds between the toxin and the channel are shown as yellow dashed lines and are specified in the right panel. The $Na_V1.4$ channel surface (molecular surface) is illustrated in gray, the selectivity filter motif (DEKA) is highlighted in red. The toxin is shown with

side-chain atoms present (coloring scheme: carbon – cyan, nitrogen – blue, oxygen – red, sulfur – green, backbone – isomer 12: orange, 13: light orange, 4: yellow).

Table S1. Protecting group strategy used for the preparation of μ-PIIIA mutants.

Isomer	Ser mutation	Connectivity	Trt[a]	Acm[a]
**16**	C11, C22S	$-C_4-C_5---S_{11}---C_{16}---C_{21}-S_{22}-$	C5-C21	C4-C16
**17**	C4S, C16S	$-S_4-C_5---C_{11}---S_{16}---C_{21}-C_{22}-$	C5-C21	C11-C22
**18**	C5S, C21S	$-C_4-S_5---C_{11}---C_{16}---S_{21}-C_{22}-$	C4-C16	C11-C22

[a]Protecting group used for the given cysteine pair.

Table S2. Analytical characterization of μ-PIIIA mutants produced in this study.

Isomer	t_R (C18) [min]	t_R (C8) [min]	MW (calc.) [mono]	MW (found) $[M+H]^+$
**16**	26.7[a]	26.3[a]	2573.2	2574.2
**17**	25.3[a]	24.4[a]	2573.2	2574.2
**18**	24.8[a]	24.0[a]	2573.2	2574.2

t_R, retention time; MW, molecular weight; calc., calculated.
[a] HPLC elution was carried out using a gradient of 0-40% acetonitrile containing 0.1% TFA (eluent B) in 40 min and 0.1% TFA in water (eluent A).

Table S3. Mean backbone RMSD values from the 400-ns MD simulation of µ-PIIIA disulfide isomers.

Isomer	Connectivity	Run 1 (Å)	Run 2 (Å)	Mean (Å)
PIIIA 1	$-C_4-C_5---C_{11}---C_{16}---C_{21}-C_{22}-$	4.90 ± 0.7	4.1 ± 0.4	4.5 ± 0.69
PIIIA 2	$-C_4-C_5---C_{11}---C_{16}---C_{21}-C_{22}-$	2.4 ± 0.4	2.7 ± 0.5	2.55 ± 0.47
PIIIA 3	$-C_4-C_5---C_{11}---C_{16}---C_{21}-C_{22}-$	7.4 ± 0.7	6.6 ± 0.6	7 ± 0.76
PIIIA 4	$-C_4-C_5---C_{11}---C_{16}---C_{21}-C_{22}-$	6.7 ± 0.6	6.5 ± 0.9	6.6 ± 0.77
PIIIA 5	$-C_4-C_5---C_{11}---C_{16}---C_{21}-C_{22}-$	7.8 ± 0.9	7.4 ± 1.2	7.6 ± 1.07
PIIIA 6	$-C_4-C_5---C_{11}---C_{16}---C_{21}-C_{22}-$	6.6 ± 0.8	5.7 ± 1.0	6.15 ± 1.01
PIIIA 7	$-C_4-C_5---C_{11}---C_{16}---C_{21}-C_{22}-$	4.2 ± 0.6	3.6 ± 0.4	3.9 ± 0.59
PIIIA 8	$-C_4-C_5---C_{11}---C_{16}---C_{21}-C_{22}-$	2.3 ± 0.1	5.0 ± 0.4	3.65 ± 1.38
PIIIA 9	$-C_4-C_5---C_{11}---C_{16}---C_{21}-C_{22}-$	4.7 ± 0.9	6.1 ± 0.6	5.4 ± 1.03
PIIIA 10	$-C_4-C_5---C_{11}---C_{16}---C_{21}-C_{22}-$	3.3 ± 0.6	2.3 ± 0.4	2.8 ± 0.71
PIIIA 11	$-C_4-C_5---C_{11}---C_{16}---C_{21}-C_{22}-$	4.9 ± 0.6	5.1 ± 0.2	5 ± 0.45
PIIIA 12	$-C_4-C_5---C_{11}---C_{16}---C_{21}-C_{22}-$	2.4 ± 0.3	2.6 ± 0.6	2.5 ± 0.48
PIIIA 13	$-C_4-C_5---C_{11}---C_{16}---C_{21}-C_{22}-$	4.7 ± 0.8	4.6 ± 0.5	4.65 ± 0.66
PIIIA 14	$-C_4-C_5---C_{11}---C_{16}---C_{21}-C_{22}-$	3.0 ± 0.3	2.6 ± 0.1	2.8 ± 0.30
PIIIA 15	$-C_4-C_5---C_{11}---C_{16}---C_{21}-C_{22}-$	5.6 ± 0.6	5.3 ± 0.9	5.45 ± 0.77

Table S4. Backbone RMSD based single-linkage clustering analysis of the µ-PIIIA disulfide isomers.

Isomer	Connectivity	No. of clusters Run 1	No. of clusters Run 2	% Trajectory represented by the largest cluster
PIIIA 1	$-C_4-C_5---C_{11}---C_{16}---C_{21}-C_{22}-$	23	134	99.28
PIIIA 2	$-C_4-C_5---C_{11}---C_{16}---C_{21}-C_{22}-$	6	9	99.94
PIIIA 3	$-C_4-C_5---C_{11}---C_{16}---C_{21}-C_{22}-$	447	1171	22.61
PIIIA 4	$-C_4-C_5---C_{11}---C_{16}---C_{21}-C_{22}-$	1975	2131	43.38
PIIIA 5	$-C_4-C_5---C_{11}---C_{16}---C_{21}-C_{22}-$	1039	709	77.50
PIIIA 6	$-C_4-C_5---C_{11}---C_{16}---C_{21}-C_{22}-$	78	870	98.97
PIIIA 7	$-C_4-C_5---C_{11}---C_{16}---C_{21}-C_{22}-$	54	16	91.85
PIIIA 8	$-C_4-C_5---C_{11}---C_{16}---C_{21}-C_{22}-$	4	36	96.74
PIIIA 9	$-C_4-C_5---C_{11}---C_{16}---C_{21}-C_{22}-$	2357	1131	42.10
PIIIA 10	$-C_4-C_5---C_{11}---C_{16}---C_{21}-C_{22}-$	4	2	99.99
PIIIA 11	$-C_4-C_5---C_{11}---C_{16}---C_{21}-C_{22}-$	109	45	98.20
PIIIA 12	$-C_4-C_5---C_{11}---C_{16}---C_{21}-C_{22}-$	2	1	100.00
PIIIA 13	$-C_4-C_5---C_{11}---C_{16}---C_{21}-C_{22}-$	60	44	84.10
PIIIA 14	$-C_4-C_5---C_{11}---C_{16}---C_{21}-C_{22}-$	2	1	100.00
PIIIA 15	$-C_4-C_5---C_{11}---C_{16}---C_{21}-C_{22}-$	641	801	86.42

Table S5. Mean molecular electrostatic potential (MEP) calculated for the μ-PIIIA disulfide isomers from their MD trajectories.

Isomer	Connectivity	Mean MEP (kJ/mol)
PIIIA 1	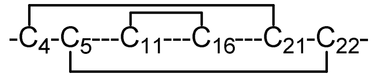 $-C_4-C_5---C_{11}---C_{16}---C_{21}-C_{22}-$	8.54
PIIIA 2	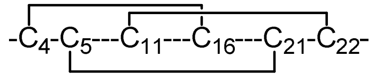 $-C_4-C_5---C_{11}---C_{16}---C_{21}-C_{22}-$	7.35
PIIIA 3	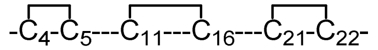 $-C_4-C_5---C_{11}---C_{16}---C_{21}-C_{22}-$	10.18
PIIIA 4	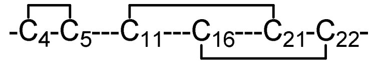 $-C_4-C_5---C_{11}---C_{16}---C_{21}-C_{22}-$	9.32
PIIIA 5	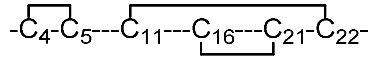 $-C_4-C_5---C_{11}---C_{16}---C_{21}-C_{22}-$	9.23
PIIIA 6	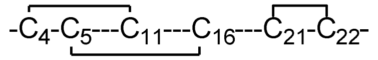 $-C_4-C_5---C_{11}---C_{16}---C_{21}-C_{22}-$	7.01
PIIIA 7	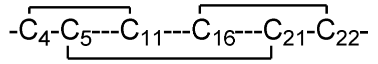 $-C_4-C_5---C_{11}---C_{16}---C_{21}-C_{22}-$	11.49
PIIIA 8	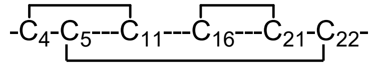 $-C_4-C_5---C_{11}---C_{16}---C_{21}-C_{22}-$	7.29
PIIIA 9	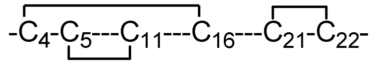 $-C_4-C_5---C_{11}---C_{16}---C_{21}-C_{22}-$	11.39
PIIIA 10	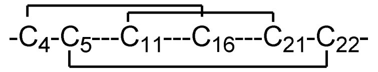 $-C_4-C_5---C_{11}---C_{16}---C_{21}-C_{22}-$	6.76
PIIIA 11	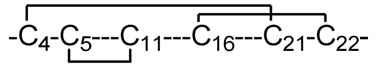 $-C_4-C_5---C_{11}---C_{16}---C_{21}-C_{22}-$	11.33
PIIIA 12	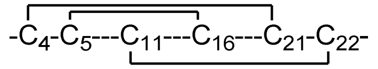 $-C_4-C_5---C_{11}---C_{16}---C_{21}-C_{22}-$	7.83
PIIIA 13	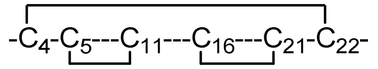 $-C_4-C_5---C_{11}---C_{16}---C_{21}-C_{22}-$	7.42
PIIIA 14	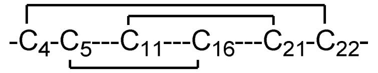 $-C_4-C_5---C_{11}---C_{16}---C_{21}-C_{22}-$	9.45
PIIIA 15	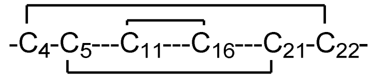 $-C_4-C_5---C_{11}---C_{16}---C_{21}-C_{22}-$	10.11

Table S6. Mean ΔG_{solv} values from the 400-ns MD simulation of μ-PIIIA disulfide isomers.

Isomer	Connectivity	Run 1 (kJ/mol)	Run 2 (kJ/mol)	Mean (kJ/mol)
PIIIA 1	$-C_4-C_5---C_{11}---C_{16}---C_{21}-C_{22}-$	2.50 ± 2.56	2.38 ± 2.69	2.44 ± 2.62
PIIIA 2	$-C_4-C_5---C_{11}---C_{16}---C_{21}-C_{22}-$	-1.73 ± 2.04	-0.64 ± 2.37	-1.18 ± 2.27
PIIIA 3	$-C_4-C_5---C_{11}---C_{16}---C_{21}-C_{22}-$	5.31 ± 2.75	6.89 ± 3.65	6.10 ± 3.32
PIIIA 4	$-C_4-C_5---C_{11}---C_{16}---C_{21}-C_{22}-$	4.49 ± 3.37	3.22 ± 2.65	3.85 ± 3.09
PIIIA 5	$-C_4-C_5---C_{11}---C_{16}---C_{21}-C_{22}-$	2.60 ± 3.19	4.73 ± 2.71	3.66 ± 3.14
PIIIA 6	$-C_4-C_5---C_{11}---C_{16}---C_{21}-C_{22}-$	8.24 ± 2.19	6.13 ± 3.54	7.18 ± 3.12
PIIIA 7	$-C_4-C_5---C_{11}---C_{16}---C_{21}-C_{22}-$	4.08 ± 2.33	5.30 ± 2.10	4.69 ± 2.30
PIIIA 8	$-C_4-C_5---C_{11}---C_{16}---C_{21}-C_{22}-$	-2.54 ± 1.78	1.82 ± 2.35	-0.36 ± 3.01
PIIIA 9	$-C_4-C_5---C_{11}---C_{16}---C_{21}-C_{22}-$	4.51 ± 2.95	5.88 ± 3.57	5.19 ± 3.34
PIIIA 10	$-C_4-C_5---C_{11}---C_{16}---C_{21}-C_{22}-$	2.33 ± 2.17	2.71 ± 2.35	2.80 ± 0.71
PIIIA 11	$-C_4-C_5---C_{11}---C_{16}---C_{21}-C_{22}-$	1.64 ± 2.30	3.74 ± 2.06	2.69 ± 2.42
PIIIA 12	$-C_4-C_5---C_{11}---C_{16}---C_{21}-C_{22}-$	2.33 ± 2.17	0.98 ± 2.10	1.65 ± 2.23
PIIIA 13	$-C_4-C_5---C_{11}---C_{16}---C_{21}-C_{22}-$	-0.30 ± 2.48	2.17 ± 2.83	0.93 ± 2.93
PIIIA 14	$-C_4-C_5---C_{11}---C_{16}---C_{21}-C_{22}-$	2.88 ± 2.49	4.71 ± 1.86	3.79 ± 2.38
PIIIA 15	$-C_4-C_5---C_{11}---C_{16}---C_{21}-C_{22}-$	5.93 ± 2.38	4.09 ± 3.14	5.01 ± 3.76

Table S7. Summary of the HADDOCK docking runs. For each isomer docking, the three best-scoring clusters are shown, ranked according to their Z-scores.

Isomer	Z-score	HADDOCK score	Cluster size	RMSD from the overall lowest-energy structure [Å]
**1**	-1.4	235 ± 13	35	1.4 ± 0.1
	-1.3	239 ± 7	37	2.2 ± 0.0
	-0.7	253 ± 27	19	0.5 ± 0.3
**2**	-1.4	399 ± 11	15	0.7 ± 0.0
	-0.8	427 ± 11	50	2.7 ± 0.0
	-0.4	447 ± 5	12	1.9 ± 0.2
**4**	-1.7	178 ± 14	41	1.5 ± 0.1
	-1.4	189 ± 21	4	1.2 ± 0.1
	-0.5	221 ± 17	7	1.9 ± 0.0
**7**	-1.6	226 ± 16	37	0.6 ± 0.4
	-0.9	249 ± 25	7	2.0 ± 0.0
	-0.6	255 ± 32	7	1.5 ± 0.2
**11**	-1.8	249 ± 23	23	0.5 ± 0.3
	-1.2	263 ± 5	56	1.1 ± 0.2
	-1.1	265 ± 14	26	2.3 ± 0.1
**12**	-1.7	213 ± 23	20	0.5 ± 0.3
	-0.7	249 ± 13	8	1.3 ± 0.0
	-0.7	250 ± 12	8	2.0 ± 0.1
**13**	-1.7	223 ± 40	7	2.1 ± 0.0
	-1.1	241 ± 10	18	1.5 ± 0.0
	-0.2	263 ± 10	29	2.7 ± 0.1
**14**	-2.2	287 ± 3	17	2.4 ± 0.1
	-0.6	315 ± 11	7	2.6 ± 0.0
	-0.5	316 ± 18	7	2.6 ± 0.2
**15**	-1.3	213 ± 14	13	2.0 ± 0.1
	-1.2	215 ± 22	40	0.5 ± 0.3
	-0.5	250 ± 13	48	1.5 ± 0.1

Table S8. Comparison of mean backbone RMSD, Rg and SASA between the unbound and Nav1.4-bound forms of the μ-PIIIA disulfide isomers 2, 7 and 15 from their respective 400-ns MD simulations

Isomer	Connectivity	RMSD (Å)	Rg (Å)	SASA (Å^2)
**2**	-C_4-C_5---C_{11}---C_{16}---C_{21}-C_{22}-	**Unbound:**<br>2.55 ± 0.47<br>**Bound:**<br>4.01 ± 0.36	**Unbound:**<br>6.99 ± 1.07<br>**Bound:**<br>8.24 ± 0.27	**Unbound:**<br>2479.51 ± 90.61<br>**Bound:**<br>2307.65 ± 89.42
**7**	-C_4-C_5---C_{11}---C_{16}---C_{21}-C_{22}-	**Unbound:**<br>3.90 ± 0.59<br>**Bound:**<br>1.69 ± 0.81	**Unbound:**<br>7.93 ± 1.42<br>**Bound:**<br>8.55 ± 0.81	**Unbound:**<br>2494.15 ± 62.60<br>**Bound:**<br>2387.73 ± 92.67
**15**	-C_4-C_5---C_{11}---C_{16}---C_{21}-C_{22}-	**Unbound:**<br>5.45 ± 0.77<br>**Bound:**<br>4.36 ± 0.29	**Unbound:**<br>10.02 ± 0.37<br>**Bound:**<br>10.01 ± 0.65	**Unbound:**<br>3166.80 ± 40.56<br>**Bound:**<br>2519.97 ± 209.36

Table S9. Mean values of the Poisson-Boltzmann binding energies (E_{bind}) between the Nav1.4-bound forms of the μ-PIIIA disulfide isomers 2, 7 and 15 from their respective 400-ns MD simulations

Isomer	Connectivity	(E_{bind}) (kJ/mol)
**2**	-C_4-C_5---C_{11}---C_{16}---C_{21}-C_{22}-	-99.56
**7**	-C_4-C_5---C_{11}---C_{16}---C_{21}-C_{22}-	-322.83
**15**	-C_4-C_5---C_{11}---C_{16}---C_{21}-C_{22}-	-144.71

Table S10. Mean values of the per residue $SASA_{res}$ compared between the unbound and Nav1.4-bound μ-PIIIA disulfide isomers 2, 7 and 15 from their respective 400-ns MD simulations

Residue	PIIIA 2 (Å^2) (Unbound)	PIIIA 2 (Å^2) (Bound)	PIIIA 7 (Å^2) (Unbound)	PIIIA 7 (Å^2) (Bound)	PIIIA 15 (Å^2) (Unbound)	PIIIA 15 (Å^2) (Bound)
Z1	260.20	243.40	264.15	247.08	266.60	247.00
R2	318.66	316.64	325.73	323.45	320.60	317.07
L3	280.91	274.57	277.72	280.49	274.03	283.27
C4	242.89	239.76	240.79	241.46	237.92	236.91
C5	240.60	239.47	241.72	234.96	242.70	242.12
G6	177.42	175.86	174.70	174.11	176.01	174.34
F7	315.56	315.25	315.70	317.76	306.89	313.87
O8	246.26	245.34	244.08	248.61	245.19	245.40
K9	295.61	299.17	299.56	301.17	287.59	296.52
S10	212.07	211.14	207.37	213.82	209.50	211.41
C11	241.55	239.86	239.75	243.75	239.07	240.48
R12	321.72	321.52	322.82	323.47	324.02	326.79
S13	209.69	231.56	208.15	210.21	207.56	210.69
R14	318.98	312.15	322.97	318.67	318.58	318.00
Q15	260.22	273.49	270.68	273.73	258.91	268.32
C16	241.55	240.88	241.06	240.12	238.77	238.06
K17	278.61	290.17	301.23	303.12	296.11	295.56
O18	247.81	245.67	245.62	247.33	247.93	243.14
H19	283.93	283.68	288.63	283.64	285.58	284.68
R20	324.54	315.74	325.48	324.41	314.86	311.76
C21	241.06	240.54	240.92	239.22	241.34	241.51
C22	240.28	242.06	239.29	242.15	243.38	242.51
